# Who Uses the Internet as a Source of Nutrition and Dietary Information? An Australian Population Perspective

**DOI:** 10.2196/jmir.4548

**Published:** 2015-08-26

**Authors:** Christina Mary Pollard, Claire Elizabeth Pulker, Xingqiong Meng, Deborah Anne Kerr, Jane Anne Scott

**Affiliations:** ^1^ School of Public Health Curtin University Perth Australia; ^2^ Department of Health in Western Australia East Perth Australia; ^3^ School of Medicine Flinders University Bedford Park Australia

**Keywords:** information seeking behavior, Internet, media, social, behavior, eating food habits, public health practice, nutrition, food, diet, Western

## Abstract

**Background:**

The Internet contains a plethora of nutrition information. Health organizations are increasingly using the Internet to deliver population-wide health information and interventions. Effective interventions identify their target population and their needs; however, little is known about use of the Internet as a source of nutrition information.

**Objective:**

The aim was to assess the change in prevalence and demographic characteristics of Western Australian adults accessing the Internet as a source of nutrition information and identify specific information needs.

**Methods:**

Data were pooled from the Western Australian Department of Health’s 3-yearly Nutrition Monitoring Survey Series telephone survey between 1995 and 2012 of 7044 participants aged 18 to 64 years. Outcome variables were the main sources of nutrition information used in the last year and yes/no responses to 4 suggestions to what would make it easier to eat a healthy diet. Sociodemographic variables were collected.

**Results:**

The proportion of respondents using the Internet for nutrition information increased from <1% in 1995-2001 to 9.1% in 2004 and 33.7% in 2012. Compared to 2004, logistic regression showed that the odds of using the Internet for this information increased significantly in 2009 (OR 2.84, 95% CI 2.07-3.88) and 2012 (OR 5.20, 95% CI 3.86-7.02, *P*<.001). Respondents using the Internet as a source were more likely to be female (OR 1.30, 95% CI 1.05-1.60, *P*=.02), live in a metropolitan area (OR 1.26, 95% CI 1.03-1.54, *P*=.03), born in countries other than Australia/UK/Ireland (OR 1.41, 95% CI 1.07-1.85, *P*=.02), more educated (university: OR 2.46, 95% CI 1.77-3.42, *P*<.001), and were less likely to be older (55-64 years: OR 0.38, 95% CI 0.25-0.57, *P*<.001). The majority of respondents agreed the following information would assist them to make healthier choices: more ways to prepare healthy foods (72.0%, 95% CI 70.7-73.3), quicker ways to prepare healthy foods (79.0%, 95% CI 77.8-80.1), how to choose healthy foods (68.8%, 95% CI 67.5-70.1), and knowing more about cooking (54.7%, 95% CI 53.3-56.1). Those using the Internet for nutrition information were more likely than nonusers to want to know quicker ways to prepare healthy foods (83.0% vs 78.1%, *P*=.005) and information on choosing healthy foods (76.3% vs 67.3%, *P*<.001).

**Conclusions:**

Use of the Internet as a main source of nutrition information has grown rapidly since 2004; one-third of Western Australian adults reported using the Internet for this purpose in 2012. Information on preparing healthy foods (ideas, quicker ways), choosing ingredients, and knowing more about cooking would make it easier to eat a healthy diet. For Internet users, emphasis should be on quicker ways and choosing ingredients. These finding have implications for policy makers and practitioners and suggest that traditional health promotion tactics should continue to be used to reach the broader population.

## Introduction

Use of technology for communication has increased and traditional sources of information have changed in importance over time. Use of the Internet in Australian homes has risen dramatically from 3.4% of households in 1996 to 80% in 2012 [1,2]. Searching for health information is the third most popular use of the Internet [3]. Recent research in the United States, Norway, and Canada estimates that almost half of Internet users search for information on diet and nutrition [4-7]. The Norwegian research estimated that 40% of online health seekers are willing to change their diet or other lifestyle aspects [5]. The size and scale of the Internet raises concerns about the credibility and reliability of the nutrition and dietary information available [8].

In Australia, diet is a significant factor contributing to the burden of disease [9], accounting for 43% of the total risk factor burden [10] at an estimated cost of AUS $67 billion per year in 2010 [11]. The general population is bombarded with misleading or inaccurate nutrition messages [12]. Population-wide health interventions aim to improve the quality and quantity of dietary choices by providing sound nutrition and dietary information. Interventions that provide accurate and balanced information enable the target population to make well-informed, healthy choices [13]. The World Health Organization encourages countries to develop food-based dietary guidelines and most nutrition interventions are based on these [14]. Australian Dietary Guidelines have provided evidence-based recommendations for a healthy diet over several decades [15-18]. The challenge for health authorities is to promote changes in dietary intake, replacing eating patterns predominated by energy-dense nutrient-poor “discretionary” foods with eating patterns that are in-line with the dietary guidelines [19].

Online health interventions have the capacity to influence voluntary behavior change and have the advantage of lower costs and increased reach when compared with more traditional channels, such as print media, with similar impact [20]. In addition, the Internet offers the ability to individually tailor intervention messages or provide interactivity that is not available with other channels [21]. Web 2.0 features have been demonstrated to have great potential for Internet-based health interventions [21]. Social media apps utilizing Web 2.0 allow for the creation and exchange of user-generated content [22] that moves beyond one-directional read-only website content [23]. More than half of US adults use social media [24] and two-thirds of Australian adults who used the Internet at home went online for social networking [2]. A third of online health seekers in the United States used social media to source health-related information in 2008 [25]. Evidence suggests that people using social media for health information are more likely to read rather than create or contribute [26]. The fastest growing source of information about health and nutrition for younger adults is social networking sites such as YouTube, Facebook, and Twitter [27].

As use of the Internet has grown, the potential to reach a large number of people has made it appealing for dissemination of nutrition interventions. Government and not-for-profit health organizations increasingly use the Internet for population-wide health interventions [20,21,28]. Effective nutrition interventions require use of timely and appropriate delivery channels for dissemination. An understanding of the population and their current media use assists with selecting appropriate social marketing communication channels [29]. At the time of conducting this research, little was known about use of the Internet as a source of nutrition and dietary information in Australia and the types of information sought.

The primary aim of this paper was to assess the change in prevalence and demographic characteristics of those using the Internet as a source to obtain nutrition and dietary information among Western Australian adults over 2 decades. To assist with the development of population-wide nutrition interventions, the association between use of the Internet as a source of nutrition and dietary information and the respondents’ perception of what would make it easier for them to eat a healthy diet was explored.

## Methods

### Sample

The data were part of the Department of Health in Western Australia’s Nutrition Monitoring Survey Series (NMSS), which aims to investigate the nutrition knowledge, attitudes, and behaviors of Western Australian adults related to the Dietary Guidelines to prioritize and monitor the impact of nutrition interventions. Computer-assisted telephone interviews with 7044 Western Australian adults aged 18 to 64 years were conducted from July to August in 1995, 1998, 2001, 2004, 2009, and 2012. The 1995 sample was stratified by geographic area and the 1998, 2001, and 2004 samples were quota sampled by gender and geographic area. The telephone numbers were selected randomly by a computer-generated random digit dialing program. In 2009 and 2012, the samples were randomly drawn from the 2008 and 2011 Electronic White Pages for Western Australia, respectively, and stratified according to area of residence. The details of survey and sampling strategies can be found elsewhere [30].

### Outcome Measurements

The main purpose of this study was to assess change in prevalence and demographic characteristics of Western Australian adults using the Internet as a source of nutrition and dietary information. To answer the main purpose, respondents were asked an open-ended question: “In the last 12 months, what have been your main sources of nutrition and dietary information?” After first responses were given, the interviewer probed “anything else?” The main sources of nutrition information used were television advertising and programs, magazine articles, books, and the Internet; this study reports on respondents’ use of the Internet. We compared respondents who used the Internet as the main source of nutrition and dietary information with those who did not mention using the Internet. The secondary aim of the study was to explore the association between use of the Internet as a source of nutrition and dietary information and the respondents’ perception of what would make it easier for them to eat a healthy diet to assist with the development of population-wide nutrition interventions. To answer the secondary aim, respondents were asked “Which of the following would make it easier for you or your family to eat a healthy diet?” with the following options: (1) knowing more ways of preparing healthy foods, (2) knowing quicker ways of preparing healthy foods, (3) having more information to help me decide if foods are healthy, and (4) knowing more about cooking (all answered “yes” or “no”). The response options for making it easier to eat a healthy diet were identified during the development stages for the NMSS tool in which open-ended questions were used and typical responses identified. Participants’ sociodemographic information (gender, age, education, area of residence, household income, employment status, country of birth, area of residence, height, and weight) was also collected.

### Statistical Analysis

The data were collected to be representative of the Western Australian population. Data for all the years were pooled and weighted to account for sampling design and adjusted for age, sex, and geographic area to a single standard population to allow for comparisons over time. The standard population used was the 2011 estimated resident population of Western Australia [31] because it was the most recent census year.

The descriptive statistics report the prevalence and 95% confidence interval (95% CI) of using the Internet to obtain nutrition and dietary information by survey year, gender, and age. Binary logistic regression was used to assess the association between respondents using the Internet as a source of nutrition and dietary information and sociodemographic characteristics. The covariates in the full model included survey year as a measure of time, gender, age, education, household income, employment status, country of birth, area of residence (metropolitan vs nonmetropolitan), body mass index (BMI) categories, and Socioeconomic Indexes for Areas (SEIFA) quintile [32]. Variables with *P* value <.05 were retained in the final model and reported. Data from surveys administered in 2009 and 2012 were compared for Internet use by gender and age to provide a more recent analysis.

All analyses were performed using Stata 12.0 (StataCorp LP, College Station, TX, USA) under the survey module and a *P* value <.05 was regarded as statistically significant.

## Results

Between 1995 and 2012, a total of 7044 adults participated in the NMSS. Details of the sample and demographic information are shown in Table 1.

The main purpose of this study was to assess change in prevalence and demographic characteristics of Western Australian adults using the Internet as a source of nutrition and dietary information. Table 2 shows that there was a significant increase in the proportion of the population using the Internet as a source of nutrition and dietary information between 1995 (0.2%, 95% CI 0.0-1.2), 2004 (9.1%, 95% CI 7.3-11.4), and 2012 (33.7%, 95% CI 30.8-36.8, *P*<.001). Across all surveys, more females (19.6%, 95% CI 17.9-21.5) used the Internet than males (14.2%, 95% CI 12.5-16.1, *P*=.005) and no significant difference was found by age. The top 5 main sources of nutrition information in 2012 were the Internet (33.7%, 95% CI 30.8-36.8), television programs (23.7%, 95% CI 21.2-26.2), magazine articles (22.5%, 95% CI 20.1-25.0), television advertising (22.0%, 95% CI 19.6-24.6), and books (19.0%, 95% CI 16.7-21.5).

**Table 1:** Sample demographics of the Nutrition Monitoring Survey Series, Western Australia, 1995-2012.

Demographic characteristics		Survey year, n						Total, %^a^ N=7044
		1995 n=1002	1998 n=1004	2001 n=1004	2004 n=1202	2009 n=1284	2012 n=1548	
**Sex**								
	Female	631	502	502	601	830	1005	49.18
	Male	371	502	502	601	454	543	50.82
**Age group (years)**								
	18-24	119	110	118	103	71	66	15.80
	25-34	257	210	245	232	180	144	23.01
	35-44	291	305	296	333	340	377	22.41
	45-54	207	234	212	297	356	466	21.33
	55-64	128	145	133	237	337	495	17.45
**Area of residence**								
	Metropolitan	748	751	75	601	965	1011	78.33
	Remote areas	51	63	62	150	29	82	4.80
	Rural areas	203	190	18	451	290	455	16.87
**Education**								
	Less than high school	376	336	303	330	221	211	19.67
	High school	251	237	265	257	178	198	21.98
	Trade/certificate/diploma	90	95	77	177	481	632	25.47
	University degree	284	336	344	435	399	504	33.58
	Missing	1	0	1	3	5	3	0.30
**Household income (AUS $)**								
	≤$60,000	748	603	558	603	349	346	37.47
	>$60,000	174	305	340	560	814	1024	51.40
	Missing	80	96	106	39	121	178	11.13
**Employment status**								
	Currently not in paid employment	330	263	278	285	364	408	26.82
	Currently in paid employment	669	741	726	917	920	1139	73.14
	Missing	3	0	0	0	0	1	0.04
**Country of birth**								
	Australia	656	665	668	868	867	1122	68.64
	UK/Ireland	189	209	193	155	202	221	15.96
	Other countries	157	130	143	179	214	205	16.38
	Missing	0	0	0	0	1	0	0.02

^a^ Percentages were weighted for probability of sample selection and adjusted by age, sex, and geographic area to the 2011 Estimated Resident Population of Western Australia.

**Table 2:** Prevalence of using the Internet as a source to obtain nutrition and dietary information, the Nutrition Monitoring Survey Series, Western Australia, 1995-2012.

Variable		No, % (95% CI)^a^	Yes, % (95% CI)^a^	*P* ^b^
**Survey year**				<.001
	1995	99.83 (98.84, 99.98)	0.17 (0.02, 1.16)	
	1998	99.43 (98.78, 99.73)	0.57 (0.27, 1.22)	
	2001	99.84 (98.89, 99.98)	0.16 (0.02, 1.11)	
	2004	90.86 (88.62, 92.70)	9.14 (7.30, 11.38)	
	2009	77.09 (73.95, 79.96)	22.91 (20.04, 26.05)	
	2012	66.27 (63.23, 69.18)	33.73 (30.82, 36.77)	
**Gender**				<.001
	Female	80.36 (78.51, 82.09)	19.64 (17.91, 21.49)	
	Male	85.80 (83.95, 87.47)	14.20 (12.53, 16.05)	
**Age group (years)**				.21
	18-24	79.92 (74.69, 84.30)	20.08 (15.70, 25.31)	
	25-34	83.93 (81.01, 86.47)	16.07 (13.53, 18.99)	
	35-44	83.22 (81.04, 85.19)	16.78 (14.81, 18.96)	
	45-54	82.89 (80.51, 85.04)	17.11 (14.96, 19.49)	
	55-64	85.14 (82.70, 87.29)	14.86 (12.71, 17.30)	
Total		83.13 (81.83, 84.35)	16.87 (15.65, 18.17)	

^a^ Percentages were weighted for probability of sample selection and adjusted by age, sex, and geographic area to the 2011 Estimated Resident Population of Western Australia.

^b^ *P* values were derived from survey design–based Pearson chi-square test.

**Figure 1:**
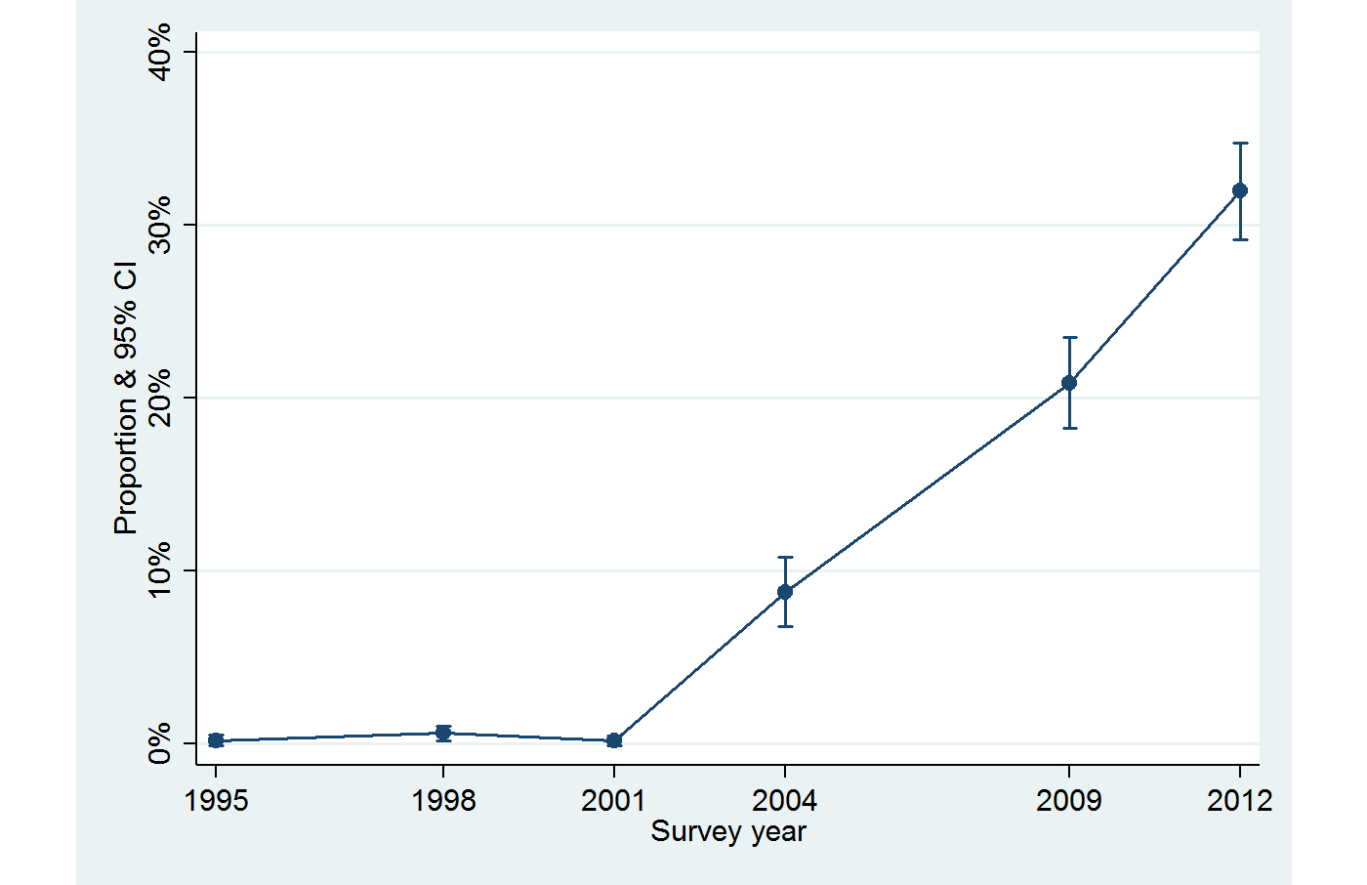
Proportion of participants using the Internet as a source of obtaining nutrition and dietary information over the survey period, the Nutrition Monitoring Survey Series, Western Australia, 1995-2012 (derived after the logistic regression).

**Figure 2:**
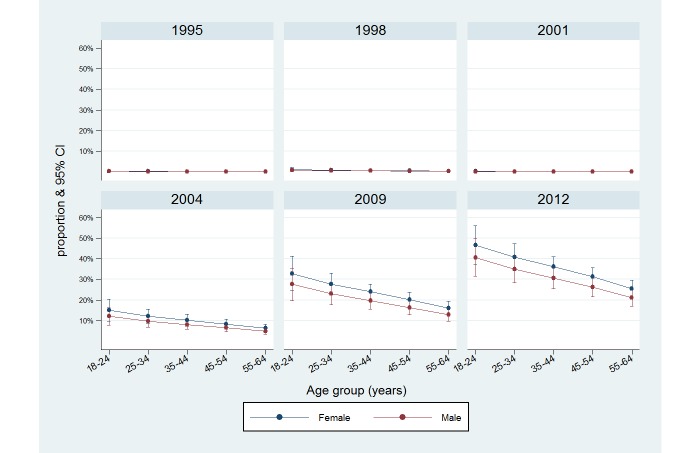
Proportion of respondents using the Internet as a source of nutrition and dietary information over the survey period by gender and age, the Nutrition Monitoring Survey Series, Western Australia, 1995-2012 (derived after logistic regression).

The logistic regression results showed a sharp increase in using the Internet for obtaining nutrition and dietary information from 2004 (Table 3 and Figure 1). After adjustment for the model covariates, gender showed a difference in the odds of using the Internet as a source. Females were more likely to use the Internet as a nutrition information source than males (OR 1.30, 95% CI 1.05-1.60, *P*=.02). Respondents living in metropolitan areas (OR 1.26, 95% CI 1.03-1.54, *P*=.03); born in countries other than Australia, United Kingdom, or Ireland (OR 1.41, 95% CI 1.07-1.85, *P*=.02); and with more than high school level education (tertiary educated: OR 2.46, 95% CI 1.77-3.42, *P*<.001) were more likely to use the Internet as a source (Table 3). Respondents aged between 35 and 64 years were less likely to use the Internet as a source compared to those aged between 18 and 24 years (*P*<.001) (Table 3 and Figure 2).

**Table 3:** Factors associated with using the Internet as a source of obtaining nutrition and dietary information, the Nutrition Monitoring Survey Series, Western Australia, 1995-2012.

Factor		OR (95% CI)	*P* ^a^
**Survey year**			<.001
	1995	0.02 (0, 0.12)	
	1998	0.06 (0.03, 0.13)	
	2001	0.02 (0.00, 0.11)	
	2004	1.00	
	2009	2.84 (2.07, 3.88)	
	2012	5.20 (3.86, 7.02)	
**Gender**			.02
	Male	1.00	
	Female	1.30 (1.05, 1.60)	
**Age group (years)**			<.001
	18-24	1.00	
	25-34	0.78 (0.51, 1.20)	
	35-44	0.64 (0.43, 0.95)	
	45-54	0.51 (0.34, 0.76)	
	55-64	0.38 (0.25, 0.57)	
**Education attainment**			<.001
	Less than high school	1.00	
	High school	1.16 (0.76, 1.76)	
	TAFE/trade/diploma	1.80 (1.29, 2.52)	
	Tertiary	2.46 (1.77, 3.42)	
**Country of birth**			.02
	Australia/UK/Ireland	1.00	
	Other countries	1.41 (1.07, 1.85)	
**Residential area**			.03
	Nonmetropolitan	1.00	
	Metropolitan	1.26 (1.03, 1.54)	

^a^ Results were derived from a binary logistic regression under survey module. *P* values were derived from Wald test.

The secondary aim of this study was to explore the association between use of the Internet as a source of nutrition and dietary information and the respondents’ perception of what would make it easier for them to eat a healthy diet to assist with the development of population-wide nutrition interventions. A high proportion of all respondents agreed that the following information would make it easier for them to eat a healthier diet: knowing more ways of preparing healthy foods (71.99%, 95% CI 70.67-73.27), knowing quicker ways of preparing healthy foods (78.96%, 95% CI 77.80-80.08), more information to help decide if foods are healthy (68.80%, 95% CI 67.49-70.07), and knowing more about cooking (54.68%, 95% CI 53.25-56.11). Table 4 shows that when compared to respondents who did not use the Internet to obtain nutrition and dietary information, a statistically significantly greater proportion of people who used the Internet agreed that knowing quicker ways of preparing healthy foods (83.03% vs 78.14%, *P*=.005) and having more information to help decide if foods are healthy (76.28% vs 67.27%, *P*<.001) would make it easier for them to eat a healthier diet.

When comparing survey results for 2009 and 2012, Table 5 shows that a significantly higher percentage of females aged between 25 and 54 years used the Internet as a source of nutrition and dietary information in 2012 with the highest users being females aged between 25 and 34 years (58.8%, 95% CI 46.4-70.1, *P*<.001). There were also higher numbers of males aged between 35 and 44 years using the Internet as a source of nutrition and dietary information in 2012 compared to 2009 (36.6%, 95% CI 27.1-47.2 vs 17.0%, 95% CI 10.8-25.8, *P*=.002).

**Table 4:** Association between using the Internet as a source to obtain nutrition and dietary information and perception of whether it would be easier for respondents to eat healthy diet.

Would make easier to eat healthy diet		Participants, % (95% CI)^a^		*P* ^b^	Total, % (95% CI)^a^
		Not using Internet	Using Internet		
**Knowing more ways of preparing healthy foods (n=7007)**				.06	
	No	28.65 (27.29, 30.05)	24.88 (21.45, 28.65)		28.01 (26.73, 29.33)
	Yes	71.35 (69.95, 72.71)	75.12 (71.35, 78.55)		71.99 (70.67, 73.27)
**Knowing quicker ways of preparing healthy foods (n=7019)**				.005	
	No	21.86 (20.65, 23.12)	16.97 (14.27, 20.07)		21.04 (19.92, 22.20)
	Yes	78.14 (76.88, 79.35)	83.03 (79.93, 85.73)		78.96 (77.80, 80.08)
**More information on healthy foods (n=6996)**				<.001	
	No	32.73 (31.35, 34.15)	23.72 (20.58, 27.18)		31.20 (29.93, 32.51)
	Yes	67.27 (65.85, 68.65)	76.28 (72.82, 79.42)		68.80 (67.49, 70.07)
**If I knew more about cooking (n=7011)**				.33	
	No	45.69 (44.19, 47.20)	43.49 (39.42, 47.64)		45.32 (43.89, 46.75)
	Yes	54.31 (52.80, 55.81)	56.51 (52.36, 60.58)		54.68 (53.25, 56.11)

^a^ Percentages were weighted for probability of sample selection and adjusted by age, sex, and geographic area to the 2011 Estimated Resident Population of Western Australia.

^b^ *P* values were derived from survey design–based Pearson chi-square test.

**Table 5:** Prevalence of using Internet as a source obtaining nutrition and dietary information, the Nutrition Monitoring Survey Series, Western Australia, 2009 and 2012.

Age range	2009 Yes, % (95% CI)^a^		*P*	2012 Yes, % (95% CI)^a^		*P*
	Female	Male		Female	Male	
18-24 years	31.58 (18.15, 49.01)	34.56 (19.05, 54.23)	.81	56.81 (38.09, 73.77)	25.80 (13.59, 43.45)	.01
25-34 years	28.40 (20.12, 38.44)	32.83 (21.07, 47.22)	.59	58.77 (46.40, 70.13)	32.67 (20.73, 47.38)	.005
35-44 years	24.19 (18.99, 30.27)	16.98 (10.76, 25.76)	.13	39.66 (33.37, 46.31)	36.57 (27.08, 47.24)	.62
45-54 years	16.91 (12.42, 22.62)	18.21 (11.58, 27.45)	.79	33.19 (27.41, 39.52)	27.11 (20.14, 35.43)	.22
55-64 years	13.48 (9.14, 19.45)	18.21 (11.64, 27.34)	0.32	23.39 (18.58, 29.01)	22.39 (16.44, 29.72)	.82

^a^ Percentages were weighted for probability of sample selection and adjusted by age, sex, and geographic area to the 2011 Estimated Resident Population of Western Australia.

## Discussion

### Prevalence and Characteristics

This study is unique because it examines characteristics of Australian adults’ use of the Internet for nutrition and dietary information and changes in usage over 2 decades. The study found dramatic growth in use of the Internet as a main source of nutrition and dietary information from 9.1% in 2004 to 33.7% in 2012 when it became the most popular main source of nutrition information. This is consistent with overall growth in Internet use in Australian homes, which increased from 3.4% in 1996 to 56% in 2004 and to 83% in 2012 [1,2,33]. However, only one-third of the Western Australian population reported using the Internet as a source of nutrition and dietary information in 2012, which was a smaller proportion than expected. A similar study in Canada found that 51% of the adult population reported using the Internet for nutrition information in 2008 [6]. The proportion of US adults using the Internet to source diet, weight, and physical activity information was 42.8% in 2011 [7]. The Pew Internet & American Life project found that 49% of Internet users had searched for information about diet and nutrition in 2006 [4] (which is equivalent to 34% of the US population [34]). There is limited Australian population research regarding sources of nutrition and dietary information. A 1999 South Australian study of 603 adults did not reveal the Internet as a commonly used source of nutrition information [35]; however, a Queensland convenience sample of 94 adults in 2013 reported that the Internet (63%) was the most utilized source of nutrition information [36]. These findings may reflect cultural differences in requirements for nutrition information. Further research is recommended to understand the implications of the differences in use of the Internet as a resource for nutrition and dietary information.

Comparison of the most recent data available from Australia, the United States, and Canada shows that a similar proportion of the adult population use the Internet overall. In Australia, 83% of adults used the Internet at home in 2012 [2], 81% of US adults used the Internet in 2012 [34], and 83% of Canadian adults used the Internet at home in 2012 [37]. In addition, most Western Australian households reported using the Internet every day (85%) and using a high-speed broadband connection (79%) [2]. A similar proportion of US households accessed the Internet at home using broadband (70%) [38,39] and almost all Canadian Internet users (97%) connected via high-speed broadband [37]. Recent statistics show 80% of US adult Internet users [3] and 70% of Canadian adult Internet users [40] searched for health information online. Improvements in search engine technology have been a key factor in increased online health information seeking over the past decade [41]. Given the rapid increase in use of the Internet in recent years in this study, it is important that governments continue to monitor the prevalence of searching for nutrition and dietary information online.

Western Australian adults using the Internet as a source of nutrition and dietary information were significantly more likely to be female; living in a metropolitan area; born in countries other than Australia, the United Kingdom, and Ireland; more educated; and younger. This is consistent with other studies from the United States, Switzerland, and Canada, which found that adults using the Internet as a source of dietary information are more likely to be female [7,35], more educated [6,7,35], and younger [6,7,35]. Canadian adults who earned a higher income were more likely to use the Internet for nutrition information [6]; however, in this study, household income was not associated with using the Internet for this purpose. Further, online seekers of general health information from the United States, France, and Germany were more likely to be of higher social class [36,42], women [42,43], and more educated [43]. Our findings reinforce the importance of sociodemographic differences when targeting and developing nutrition interventions, particularly gender and education. Reasons for these differences and implications for engagement need further exploration.

Weight status was not associated with using the Internet for nutrition and dietary information in this study. This is surprising because the Internet has been identified as an important source of information and support for individuals with stigmatized health conditions [44,45], including obesity [46]. American adults using the Internet to help with diet, weight, and physical activity were more likely to have a higher BMI [7] and Canadian adults using the Internet for nutrition information were more likely to have tried a popular diet in the past year [6]. Obese adults from Australia report using the Internet to search for information to assess their likelihood of developing a serious disease as a result of their obesity and ways to minimize the health risks, including healthy recipes and ideas to increase physical activity [45]. Use of the Internet as a source of nutrition and dietary information by obese adults remains an important area to monitor in Australia to provide input to future public health interventions.

### Internet-Based Information That Would Make it Easier to Eat Healthily

Western Australians who used the Internet as a source of nutrition and dietary information were more likely than nonusers to agree that quicker ways of preparing healthy foods and help deciding if foods are healthy would help them to eat more healthily. The majority of all respondents also agreed that information about preparing healthy foods and cooking would assist. These findings are consistent with identified barriers to healthy eating. Barriers typically include time pressures, the desire for convenience, and lack of motivation to cook, rather than a lack of skills or knowledge [47]. The US Food Hero social media nutrition intervention, which focused on the importance of serving healthy meals, aimed to provide healthy recipes that overcame time and cost barriers [48]. The US Supplemental Nutrition Assistance Program (SNAP) participants reported that the activities that would motivate them to continue to use an Internet resource for food and nutrition included recipe or product ratings, blogs, and discussion boards [49].

### Recent Survey Results

Data from the 2009 and 2012 surveys were examined to explore recent changes in prevalence and the demographic profile of Internet users to guide current nutrition program development. Results showed that more than half of females aged between 18 and 34 years used the Internet as a source of nutrition and dietary information in 2012. The percentage of female users aged between 25 and 34 years doubled between 2009 and 2012 as did the percentage of male users aged between 35 and 44 years (to 37%). These results suggest that the Internet is increasing in importance for males and females. The opposite trend was seen for males aged between 18 and 24 years. One possibility is that younger males, typically the early adopters of newer technologies, are using other sources of information (eg, smartphone apps) and this should be investigated in further research. The demographic characteristics of US Internet users were also found to change significantly between 2007 and 2011 [7].

The recent increase in use of the Internet for nutrition and dietary information by younger females aged between 18 and 34 years and males aged between 35 and 44 years shows the dynamic nature of the resource. Although the overall proportion of Western Australians using the Internet as a source was relatively lower than results of similar studies, there are subgroups emerging that rely on the Internet more heavily. Changes in the demographic characteristics of users over time is an important area to continue to monitor.

### Quality of Information

The NMSS is a government survey that contributes evidence of prevalence of use and demographic information to Western Australian policy and programs. It is critical that high-quality information is used to guide population dietary decision making. The size and scale of the Internet raises concerns about the credibility and reliability of the nutrition and dietary information available [8]. Most people use general search engines to find health information online [43] that present search results based on a page ranking system that is open to manipulation through website design [41]. Analysis of selected Canadian websites commonly used for nutrition information found that commercial websites typically contained some poor and misleading advice [50]. The Internet information most likely to be viewed for weight loss and weight management was also found to contain inaccurate information [41]. More accurate information was provided by medical, government, and university websites; however, they appeared in the second and third pages of the website searches so were less likely to be viewed [41]. Some research suggests that Internet users do not discriminate much in terms of quality of information for nutrition information [4,6,50,51]. A large proportion of online health seekers do not consistently check the source and date of the health information they find online [4] and use commercial websites to seek health and nutrition information [50]. Less than half of Canadians rated the Internet as a source of nutrition information that was very or extremely credible [6]. Recognition of health brands that are trusted in the non-Internet world have been shown to assist with identifying which Internet information to trust [51]. Health organizations need to ensure quality nutrition and dietary information is widely available and accessible on the Internet, which could include introducing certification of websites that provide trustworthy information.

### Use of the Internet for Population-Wide Nutrition Interventions

Use of the Internet as a source of nutrition and dietary information has shown recent rapid uptake by Western Australian adults making it appealing for dissemination of public health interventions. Internet-based nutrition and dietary interventions should incorporate good practice characteristics [52] including identifying and understanding the target audience [29] and then customizing and tailoring communications to meet their needs [53]. For Internet-based interventions to be effective, they need to address issues including levels of participation and adherence [22], and fully utilize the interactive nature of Web 2.0 social media platforms [23]. Organizations that choose to engage in social media should maintain content and participate regularly [29], monitor poor quality health information, and provide the credible alternative [23], which can be time consuming to manage [54]. The increasing use of the Internet by health organizations for population-wide health interventions highlights the need for guidelines for effective communication strategies, such as the toolkits from the Centers for Disease Control and Prevention in the United States [28].

In this study, the majority of the population did not report that they used the Internet as a main source of nutrition and dietary information. It is important for population-wide education, such as nutrition interventions, to be inclusive and reach the majority of the population, in this case, reaching adults that report using sources other than the Internet. Australian adults living in regional areas, older than 65 years, living alone, or with low household income are less likely to use the Internet in general [55]. Use of multiple channels as appropriate to the intervention and target population is advised [56]. Messages are reinforced when communicated across a number of channels; for instance, integrating traditional public health intervention tactics with social media [53].

Another important consideration for Internet-based nutrition and dietary interventions is the level of literacy required. Almost half of Australians aged between 15 and 74 years have literacy skills below the level deemed suitable to meet societal demands, including using the Internet [57]. For Internet-based interventions to be inclusive, they should be designed to meet the needs of those with limited literacy [23] by introducing non-text-based social media, including use of images, illustrations, video, and sound [58]. Use of traditional health intervention tactics to engage with groups identified as hard to reach via the Internet can remain effective. For example, an Australian telephone-based nutrition intervention has successfully recruited participants from more disadvantaged and regional areas [59,60]. The intervention recently incorporated an interactive website to assist with recruiting new participants and engaging existing participants [61].

### Limitations

There are strengths and limitations to the current study. The results can be generalized to Western Australia due to the high response rate and representative sample selection. Development of survey question terminology including “the Internet” was completed in 1995. Due to the recent rapid uptake of the Internet and other developments, including Web 2.0 social media platforms and smartphone technology, terminology may need to be updated in future surveys to reflect commonly used language to ensure usage is captured fully. Use of the Internet to source nutrition and dietary information has been reported for the Western Australian adult population, not of Western Australian Internet users. In future, collecting data on use of the Internet, device used, and other relevant technology may help to provide context to use of the Internet to source health and nutrition information. Frequency of use of the Internet to source nutrition and dietary information, the websites that were used, and quality of information sourced were not measured and more information is urgently needed in this area. The study does not specifically investigate the information sources used depending on the information required; for example, information sources for how to feed a toddler a healthy diet may differ to seeking advice on losing weight or healthy recipe ideas for family eating. The cross-sectional survey results are self-reported and are not validated against objective measures; however, it provides useful evidence to measure public attitudes. Height and weight measures are self-reported; however, the use of a correction formula attempted to account for possible underestimation of weight status. This is a population study but there may be differences between Western Australia and other Australian states and territories; for example, more Western Australian households are connected to broadband Internet using mobile broadband (41% vs national average of 33%) [2], which could lead to differences in use of the Internet for nutrition and dietary information. Further research is needed to explore population use of the Internet for nutrition and dietary information and how to deliver public health interventions effectively.

### Conclusions

This study found that there had been dramatic growth in using the Internet as a source of nutrition and dietary information since 2004; however, the majority of the adult population still obtain their information from other sources. Relatively fewer Western Australians used the Internet for this purpose when compared with other Western countries, but their demographic characteristics were broadly consistent. This study found that increased weight was not associated with use of the Internet as a source, which was surprising because the Internet has been identified as an important resource for individuals with stigmatized health conditions, including obesity. Given the rapid increase in use of the Internet in recent years, it is likely that prevalence of using the Internet to source nutrition and dietary information will continue to change. Changes in the prevalence and characteristics of users over time are important areas to continue to monitor to inform future development of nutrition interventions.

The Internet provides a cost-effective platform to reach the identified users with nutrition and dietary interventions, but should be integrated with traditional health promotion tactics to reach the broader population. Policy makers and practitioners delivering Internet-based nutrition interventions should ensure they identify and understand the target population, and customize and tailor communications to meet their needs. Use of non–text-based social media, including images, illustrations, video, and sound, should be included to meet the needs of those with limited literacy. Provision of credible, reliable, and practical information is recommended, including quicker ways to prepare healthy foods and how to choose healthy foods. It is also important for policy makers to improve provision of quality nutrition and dietary information on the Internet generally, which could include certification of websites that provide trustworthy information.

## Abbreviations

BMI: body mass index
NMSS: Nutrition Monitoring Survey Series
SEIFA: Socio Economic Indexes For Areas
SNAP: Supplemental Nutrition Assistance Program
